# Protective Effect of *Hericium erinaceus* on Alcohol Induced Hepatotoxicity in Mice

**DOI:** 10.1155/2015/418023

**Published:** 2015-04-16

**Authors:** Lijun Hao, Yuxi Xie, Guikai Wu, Aibin Cheng, Xiaogang Liu, Rongjuan Zheng, Hong Huo, Junwei Zhang

**Affiliations:** ^1^Department of Gastroenterology, Tangshan Gongren Hospital, Tangshan 06300, China; ^2^Department of Intensive Care Unit, Hebei United University Affiliated Hospital, Tangshan 06300, China; ^3^Department of Hepatobiliary Surgery, Tangshan Gongren Hospital, Tangshan 06300, China

## Abstract

We investigated the effects of *Hericium erinaceus* (HEM) on liver injury induced by acute alcohol administration in mice. Mice received ethanol (5 g/kg BW) by gavage every 12 hrs for a total of 3 doses. HEM (200 mg/kg BW) was gavage before ethanol administration. Subsequent serum alanine aminotransferase (ALT) level, aspartate aminotransaminase (AST) level, Maleic dialdehyde (MDA) level, hepatic total antioxidant status (TAOS), and activated nuclear factor kappa-light-chain-enhancer of activated B cells (NF-*κ*B) were determined by ELISA and immunohistochemistry, respectively. HEM administration markedly (*P* < 0.05) decreased serum ALT, AST, and MDA levels. The hepatic histopathological observations showed that HEM had a relatively significant role in mice model, which had alcoholic liver damage. In conclusion, we observed that HEM (200 mg/kg BW) supplementation could restrain the hepatic damage caused by acute alcohol exposure.

## 1. Introduction

Alcohol is the most abused substance worldwide and a significant source of liver injury [1]. Long-term alcohol consumption induces oxidative stress in the liver due to the imbalance between the prooxidant and the antioxidant systems [2]. Persistent oxidative stress results in fatty liver, which can lead to inflammation, fibrosis, cirrhosis, and even liver cancer [3]. Although important progress has been made in understanding the pathogenesis of alcoholic liver disease, the mechanisms involved in the development of the disease are not fully understood [4–6]. Thus, novel agents that correct the fundamental cellular disturbances resulting from excessive alcohol consumption are needed.

Mushrooms and primarily basidiomycetous fungi are popular and valuable foods that are low in calories and high in minerals, essential amino acids, vitamins, and fibers [7, 8]. Some of them produce substances with potential medical effects and are called medicinal mushrooms [9–12].* H. erinaceus* is a temperate mushroom that has been domesticated and is commercially grown in China. The previous study demonstrated that this mushroom exhibited cytoprotection activity against ethanol-induced gastric ulcers in rats [13]. However, the roles of HEM on alcohol induced hepatotoxicity have not been reported. Thus, the present work aims to investigate the effects of* Hericium erinaceus* (HEM) on serum alanine aminotransferase (ALT) level, aspartate aminotransaminase (AST) level, Maleic dialdehyde (MDA) level, and hepatic total antioxidant status (TAOS) in mice liver injury induced by acute alcohol administration.

## 2. Material and Methods

### 2.1. Animals

Female Kunming strain mice weighing 20–22 g were purchased from the Experimental Animal Center, Hebei University of Traditional Chinese Medicine, China. The mice were maintained at room temperature under alternating natural light/dark photoperiod and had access to standard laboratory food and fresh water* ad libitum*. All animal experiments followed the Guidelines published by the Ministry of Science and Technology of China. Care was taken to minimize discomfort, distress, and pain to the animals.

### 2.2. Preparation of* Hericium erinaceus* (HEM)

Fermented mushroom of HEM was produced in Hebei United University, China. The aqueous extraction was performed by adding 100 mL boiling water to 10 g air-dried mycelium. The infusion stood at room temperature for 30 minutes. After cooling and filtration, the extract was frozen and concentrated by lyophilization for five days overnight, in order to obtain the HEM.

### 2.3. Experimental Design

The binge drinking mouse model developed by Wertheimer et al. [14] was utilized for ethanol challenge. Nine-week-old mice were allocated equally into three groups with 10 mice in each group: HEM (200 mg/kg BW)/ethanol, ethanol treatment, and control treatment. Mice received 5 g/kg BW ethanol by gavage every 12 hrs for a total of 3 doses. Control mice received an isocaloric maltose solution. In the AbM/ethanol group, AbM was dissolved in PBS and gavaged simultaneously with EtOH at a dose of 200 mg/kg BW. After the final ethanol dose, the mice were sacrificed and the blood and livers were collected. The serum was obtained by centrifugation using a serum separator tube and the stored immediately at −20°C to estimate serum alanine aminotransferase (ALT), serum aspartate aminotransaminase (AST), and liver was used to estimate inflammatory cells and inflammatory mediators.

### 2.4. Estimation of Serum ALT and AST

Serum ALT and AST activity was measured colorimetrically using a diagnostic kit (Procedure number 505, Sigma Chemical Co., St Louis, MO) according to the instructions provided.

### 2.5. Estimation of Maleic Dialdehyde (MDA)

MDA was determined with thiobarbituric acid (TBA) using the manufacturer's instructions (Nanjing Jiancheng Bioengineering Institute). Total protein content of the samples was analyzed using coomassie blue assay (Nanjing Jiancheng Bioengineering Institute).

### 2.6. Quantification of NF-*κ*B Activity

Liver tissue extracts were obtained by homogenization of snap-frozen liver tissue in Cell Lysis Buffer, subsequent sonication, and centrifugation. Activated NF-*κ*B was quantified in liver tissue extracts via ELISA-technique using the PathScan Phospho-NF*κ*B p65 (Ser536) Sandwich ELISA Antibody Pair (Shanghai Yubo Biological Technology, Inc., China), following the manufacturer's instruction.

### 2.7. Measurement of Total Antioxidant Status

The total antioxidant status (TAOS) of liver was determined as previously described by Laight et al. [15]. The increase of absorbance at 405 nm was measured by a microplate reader (Shanghai Xunda Medical Technology, Inc., China).

### 2.8. Histological Examination of Liver

Liver samples were collected and fixed in formalin for histology study. And formalin-fixed paraffin tissue sections were processed for staining with hematoxylin and eosin and then studied by light microscopy.

### 2.9. Statistical Analysis

All data were analyzed by a one-way analysis of variance, and the differences between means were established by Duncan's multiple-range test. The data are shown as the mean ± SEM. The significant level of 5% (*P* < 0.05) was used as the minimum acceptable probability for the difference between the means.

## 3. Results

### 3.1. The Effect of HEM on Serum ALT and AST

The results of serum ALT and AST are shown in Table 1. A significant increase of ALT and AST levels was observed in the ethanol group, as compared to the control mice (*P* < 0.05). HEM (500 mg/kg) treated mice showed a significant decrease in ALT and AST levels as compared to the ethanol group (Table 1) (*P* < 0.05).

### 3.2. The Effect of HEM on MDA

The liver homogenates from control mice contained low MDA level. MDA in ethanol group was significantly higher than that of control group (*P* < 0.01). HEM (500 mg/kg) treated mice showed significantly (*P* < 0.05) decreased ethanol-induced MDA elevation in hepatic tissues (Table 2).

### 3.3. Effects of HEM on Total Antioxidant Status (TAOS)

The results of hepatic TAOS are shown in Table 3. TAOS in the ethanol group was significantly (*P* < 0.01) higher than those in the normal group. Those in the HEM (500 mg/kg) treated group were significantly lower than those in the ethanol group (*P* < 0.01).

### 3.4. The Effect of HEM on NF-*κ*B Activation

**Figure 1:**
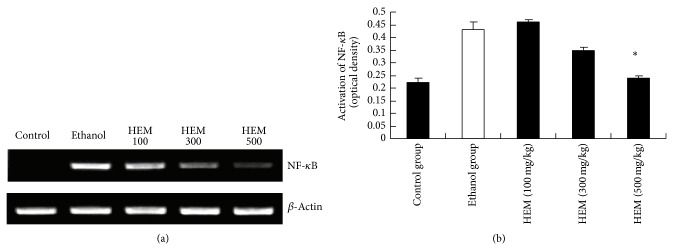
Effect of HEM on protein expression of NF-*κ*B. Values represent the mean ± SEM. ^∗^ *P* < 0.05 versus ethanol group.

As shown in Figure 1(a), protein expression of NF-*κ*B was significantly increased in the ethanol group, suggesting that ethanol induced an increase in nuclear translocation of NF-*κ*B. Conversely, protein expression of NF-*κ*B in the HEM (500 mg/kg) treated group was significantly lower than those in the ethanol group (Figure 1(b)).

### 3.5. The Effect of HEM on Hepatic Injury

**Figure 2:**
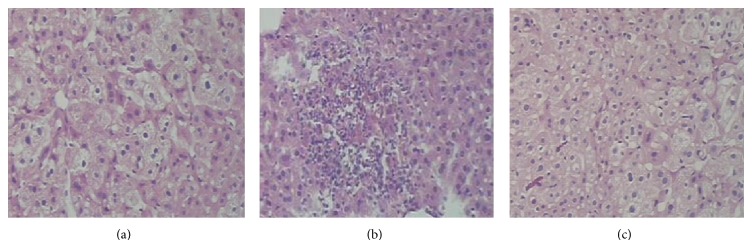
Effects of HEM on mouse liver sections using hematoxylin and eosin staining. (a) Section from a normal control mouse liver. (b) The liver section obtained from alcohol-induced mice showed a variety of cavitation and necrosis in hepatocytes. (c) Liver tissue section prepared from the HEM-treated group showed less cavitation and necrosis than (b).

On histologic analysis, ethanol treatment developed severe lymphocytes and neutrophils infiltration around the veins of hepatic tissues (Figure 2(b)). It was dramatically reduced and became more normal in HEM treated mice (Figure 2(c)).  Figure 2(a) showed that there were no cavitations, necrosis, or fibrosis in normal mice.

## 4. Discussion

In the current experiments, we used an animal model of acute binge drinking and acute ethanol toxicity showed that supplementation of HEM attenuated acute ethanol-induced liver injury. Liver damage in animals due to ingestion of ethanol is a well-known phenomenon. One sign of hepatic injury is the leakage of cellular enzymes into plasma [16]. In the present study, we have confirmed that HEM inhibited increased AST and ALT levels in serum of mice treated by ethanol. The magnitude of hepatic damage is assessed by measuring the level of released cytosolic transaminases including ALT and AST in circulation [17]. Liver function was evaluated by assessing serum ALT and AST, since AST and ALT are sensitive indicators of liver cell injury [18].

Hepatic MDA activity is commonly used as an indicator of liver tissue damage involving a series of chain reactions [19]. Induction of oxidative stress was identified as key element in the pathophysiology of liver injury induced by acute alcohol administration [20]. In this study, MDA in ethanol group was significantly higher than that of control group (*P* < 0.01). MDA level in HEM group was significantly lower than that of ethanol group (*P* < 0.05). It indicated that the free radicals being released in the liver were effectively scavenged by HEM. This result may account for the reason to inhibit ethanol toxicity.

Oxidative stress is a serious causative factor of hepatic dysfunction and plays an important role in the pathophysiology of several diseases, including atherosclerosis, diabetes, neuronal disorders, and ischaemia-reperfusion injury [21]. We measured TAOS activity as an indirect indication of oxidative stress. In the alcohol induced liver injury mouse model, we detected higher hepatic TAOS, which was decreased in the HEM-treated group. We hypothesized that HEM inhibit ethanol toxicity through decreasing the levels of TAOS activities.

NF-*κ*B is a key transcription factor in the activation of genes related to proinflammatory response. It is one of the important mechanisms linking proinflammatory response to alcoholic liver disease [22]. The present study demonstrates that alcohol induced activation of NF-*κ*B. Our results indicate that treatment with HEM suppressed alcohol induced hepatic activation of NF-*κ*B (Figure 1). Such a mechanism contributes probably to the beneficial effect of HEM on alcohol induced hepatotoxicity in mice.

Ethanol-induced hepatic injury was indicated by liver pathological changes characterized by lymphocytes and neutrophils infiltration around the veins of hepatic tissues. In the blank control group (Figure 2(a)), the hepatocytes and plate from hepatic tissue sample had an intact structure, and the boundary between hepatocytes was clear. However, the hepatocytes showed the hepatocytes' morphological damages in vein and the collection of lymphocyte and neutrophils in the ethanol control group (Figure 2(b)). Compared with the ethanol control group, Figure 2(c) showed markedly fewer cavitations and less fibrosis in the liver. These experimental phenomena indicated that HEM could weaken the liver injury caused by alcohol.

In conclusion, this study demonstrates that HEM supplementation could restrain the hepatic damage caused by acute alcohol exposure. HEM reduced damage by the inhibition of NF-kB activation and decreased the levels of TAOS activities. Hence, the present results suggest for the first time hepatoprotective effects of HEM in a model of alcoholic liver disease.

## Figures and Tables

**Table 1:** Effect of HEM on ALT and AST.

Different groups	ALT (U/L)	AST (U/L)
Ethanol group	111.0 ± 21.1	111.0 ± 11.0
Control group	31.6 ± 3.3^∗^	36.6 ± 6.8^∗^
HEM 500 group	66.6 ± 8.8^∗^	50.6 ± 8.3^∗^

Values are shown as means ± SEM, ^∗^ *P* < 0.05 versus ethanol group.

**Table 2:** Effect of HEM on MDA level.

Different groups	MDA level (*μ*mol/g)
Ethanol group	1.26 ± 0.05
Control group	0.431 ± 0.01^∗∗^
HEM 500 group	0.86 ± 0.01^∗^

Values are shown as means ± SEM, ^∗^ *P* < 0.05 versus ethanol group, ^∗∗^ *P* < 0.01 versus ethanol group.

**Table 3:** Effect of HEM on TAOS activity (*µ*M L-ascorbate).

Different groups	TAOS activity (*µ*M L-ascorbate)
Ethanol group	80.31 ± 9.30
Control group	26.46 ± 3.16^∗∗^
HEM 500 group	66.46 ± 3.22^∗^

Values are shown as means ± SEM, ^∗^ *P* < 0.05 versus ethanol group, ^∗∗^ *P* < 0.01 versus ethanol group.
